# Myelin Oligodendrocyte Glycoprotein Antibody-Associated Disease With False-Positive Results in SARS-CoV-2 Antigen Tests: A Case Report

**DOI:** 10.7759/cureus.31514

**Published:** 2022-11-14

**Authors:** Naoki Yamamoto, Hajime Ikenouchi, Yoshiki Takai, Kaoru Endo, Masashi Aoki

**Affiliations:** 1 Division of Neurology, Sendai City Hospital, Sendai, JPN; 2 Department of Neurology, Tohoku University Graduate School of Medicine, Sendai, JPN

**Keywords:** sars-cov-2 antigen test, false-positive, myelitis, sars-cov-2, myelin oligodendrocyte glycoprotein (mog)

## Abstract

A 23-year-old man presented with headache, fever, and urinary retention. Severe acute respiratory syndrome coronavirus 2 (SARS-CoV-2) antigen tests were positive, but SARS-CoV-2 polymerase chain reaction (PCR) results were negative. MRI showed long spinal cord lesions. Due to positive serum and cerebrospinal fluid myelin oligodendrocyte glycoprotein (MOG) antibodies, we made the diagnosis of MOG-associated disease. We concluded that the antigen tests were false positives because SARS-CoV-2 IgM and IgG were not elevated. Although the mechanism behind the false-positive results is unclear, physicians should consider the possibility of a false-positive result in the SARS-CoV-2 antigen test.

## Introduction

Myelin oligodendrocyte glycoprotein (MOG) antibodies have been reported to cause various central nervous system inflammatory demyelinating diseases, and MOG antibody-associated disease (MOG-AD) sometimes presents with infection-like symptoms. Given the recent spread of severe acute respiratory syndrome coronavirus 2 (SARS-CoV-2) infection in recent years, cases of MOG-AD that developed after SARS-CoV-2 infection have been reported [1,2]. Furthermore, SARS-CoV-2 infection itself induces central nervous system disorders such as encephalitis [3]. Therefore, discrimination between MOG-AD and SARS-CoV-2 infection is essential. The antigen test is widely used as a rapid tool for the diagnosis of SARS-CoV-2 infection because of its high specificity. However, we experienced a case of MOG-AD with false-positive results in SARS-CoV-2 antigen tests.

## Case presentation

**Figure 1:**
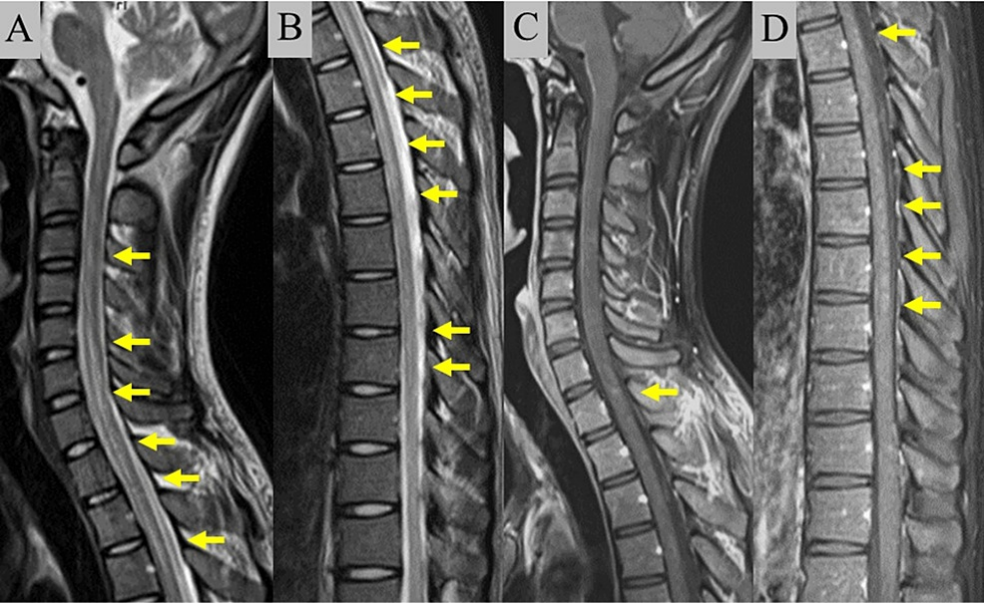
Diagnostic findings of myelin oligodendrocyte glycoprotein antibody-associated disease (MOG-AD). A and B: T2-weighted imaging of the spinal cord revealed a long lesion in the C2-T8 region (arrow). C and D: Gadolinium-enhanced T1-weighted imaging revealed a partially enhanced lesion in the C2-T8 region (arrow).

A 23-year-old healthy man presented to our hospital complaining of fever and headache for 11 days, urinary retention for six days, and a numb feeling in the trunk on the same day. He was admitted to our hospital on the 12th day after the onset of symptoms. He had no prior history of infection or vaccination. Neurological examination showed neck rigidity, hyperreflexia in the extremities with Babinski reflexes, spasticity in the right upper extremity, numbness below the bilateral T7 level, and urinary retention. Although he had no symptoms of upper respiratory tract infection, SARS-CoV-2 could not be ruled out due to the presence of low fever, and we performed the SARS-CoV-2 antigen test three times, on the second, sixth, and ninth day after symptom onset. The test results on the sixth and ninth days were positive, suggesting SARS-CoV-2 infection. However, all SARS-CoV-2 polymerase chain reaction (PCR) tests, which were performed on the sixth, ninth, and 12th day after onset (real-time PCR for the first and second tests and isothermal nucleic acid amplification for the third test), were negative. PCR tests for influenza A and B, adenovirus, respiratory syncytial virus, human metapneumovirus, and mycoplasma were also negative (isothermal nucleic acid amplification). Blood test data also showed no findings suggestive of infection, and chest CT showed no pneumonia. On the other hand, spinal cord MRI showed a long lesion from C2 to T8 with high intensity on T2-weighted imaging, with partial enhancement on gadolinium-enhanced T1-weighted imaging (Figure 1A, 1B, 1C, 1D). Cerebrospinal fluid examination showed pleocytosis with mononuclear cell predominance (cell count 209/μl, mononuclear cells 98%, polynuclear cells 2%. normal value of cell count ≤5/µl [4]). We searched for autoantibodies in the serum and cerebrospinal fluid. Serum anti SS-A, SS-B, and deoxyribonucleic acid antibody were all negative, and serum angiotensin-converting enzyme was at a normal level. Aquaporin-4 (AQP4) antibody measured with a cell-based assay was negative, but MOG antibody measured with a cell-based assay was positive in both serum (2,048-fold) and cerebrospinal fluid (8-fold) [5]. Based on these findings, we diagnosed him with MOG-AD, and inferred that the SARS-CoV-2 antigen tests were false positives. His fever and neurological symptoms gradually improved with supportive care. The spinal cord lesions shrunk to the C7 level, and the contrast enhancement disappeared on MRI on the 27th and 28th day after onset. He was discharged on the 31st day of the disease without any sequelae. One month later, we confirmed that SARS-CoV-2 IgM and IgG measured with chemiluminescent immunoassay were not elevated in the patient’s serum, and we concluded that the results of the SARS-CoV-2 antigen tests were false positives.

## Discussion

Although several reports have suggested an association between SARS-CoV-2 infection and the onset of MOG-AD [1,2], there have been no reports of false positive results in the SARS-CoV-2 antigen test at the diagnosis of MOG-AD.

We considered three possible mechanisms of the false positive results. First, the patient was actually infected with SARS-CoV-2, which could have triggered MOG-IgG production. However, this seems less likely because of the negative PCR swabs; IgM and IgG serology for SARS CoV-2 in this patient were all negative. Second, there was a problem with the quality of antigen test kits or the technical problems of the test, e.g., contamination during the test by the examiner or errors during specimen transport. But the antigen test was performed twice at different facilities, making it difficult to accept the same error was repeated. Moreover, the antigen tests were performed using an immunochromatography kit (QuickNavi™-COVID19 Ag [Denka Co., Ltd., Tokyo, Japan]), which is reported to have high specificity [6]. One retrospective study reported seven false positives on this test kit out of 2721 cases (false positivity: 0.27%), with no apparent cause identified [7]. Therefore, considering the quality of the kit, repeated false positive results are unlikely to occur. The final possibility is cross-reaction. Cross-reactivity with human rhinovirus or HIV may be the cause of false-positive results using the same technique, but the details are unclear [8,9]. One possible explanation for the false-positive results in our case is the presence in the nasopharynx of a virus with a common antigen with SARS-CoV-2. However, to the extent of our testing, we could not identify any obvious viral infections. Another possible mechanism is that the MOG antibody leaking from the serum into the nasopharyngeal swab may have cross-linked with the antibody in the test kit. However, due to the favorable clinical course of this case, we did not perform a post-discharge follow-up of the MOG antibody and antigen testing for SARS-CoV-2. Therefore, this hypothesis could not be validated. In addition, the antigen test kit for SARS-CoV-2 we used in this case was designed to analyze the nasopharyngeal swabs, making it difficult to study with serum or cerebrospinal fluid that was confirmed positive for MOG antibodies. These are the limitations of this case and further studies should be warranted.

## Conclusions

This is a case of MOG-AD in which the SARS-CoV-2 antigen test was repeatedly false-positive. Differentiating MOG-AD from infectious diseases including SARS-CoV-2 is important because MOG-AD can show symptoms similar to those of infections, such as headache and fever. If a patient presents with infectious and neurological symptoms and the SARS-CoV-2 antigen test is positive, there are three possibilities. First, the SARS-CoV-2 infection directly causes neurological damage. Second, the SARS-CoV-2 infection triggers some neurological disease. Third, the SARS-CoV-2 antigen test is false positive and the patient has another neurological disease.

The presence or absence of SARS-CoV-2 infection is critically important in determining the course of treatment strategies. Although the details of the mechanism by which SARS-CoV-2 antigen tests show false-positive results are unknown, physicians should consider the possibility of a false-positive test result and make an appropriate diagnosis based on the overall test results and clinical presentation.
